# Managing uncertainty in medicine quality in Ghana: The cognitive and affective basis of trust in a high-risk, low-regulation context

**DOI:** 10.1016/j.socscimed.2019.112369

**Published:** 2019-08

**Authors:** Heather Hamill, Kate Hampshire, Simon Mariwah, Daniel Amoako-Sakyi, Abigail Kyei, Michele Castelli

**Affiliations:** a Department of Sociology, Oxford University, 42-43 Park End Street, Oxford, OX1 1JD, UK; b Department of Anthropology, Durham University, Dawson Building, South Road, Durham, DH1 3LE, UK; c Department of Geography and Regional Planning, University of Cape Coast, Cape Coast, Ghana; d Medical School, University of Cape Coast, Cape Coast, Ghana; e The Ghana College of Nurses and Midwives**,** Pentecost University**,** Accra, Ghana; f Institute of Health and Society, Newcastle University The Baddiley-Clark Building, Richardson Road, Newcastle upon Tyne, NE2 4AX, UK

**Keywords:** Ghana, Trust, Uncertainty, Medicine quality, Medicine supply chains, Ethnography, Risk

## Abstract

Where regulation is weak, medicine transactions can be characterised by uncertainty over the drug quality and efficacy, with buyers shouldering the greater burden of risk in exchanges that are typically asymmetric. Drawing on in-depth interviews (N = 220) and observations of medicine transactions, plus interviews with regulators (N = 20), we explore how people in Ghana negotiate this uncertainty and come to *trust* a medicine *enough* to purchase or ingest it. We identify two mechanisms – attempts to mitigate uncertainty through seeking observable signs of quality and attempts to reduce informational asymmetry – that underpin cognitive assessments of a medicine's trustworthiness. However, these ‘cognitive’ forms of trust assessment have limited traction where uncertainty is high and trustworthiness remains unknowable, so a third mechanism comes into play: one based on affective relationships within which transactions are socially embedded. Even these, however, cannot eliminate uncertainty, because of the dispersed and under-regulated nature of wider supply chains. In conclusion, we reflect on the need for careful research on actors' practices and decision-making across supply chains to inform more effective policy and regulation.

## Highlights

• Weakly regulated medicine supply chains create significant trust issues.
• Buyers must negotiate uncertainty and come to *trust* a medicine *enough* to purchase or ingest it.
• Cognitive and affective based assessments of trustworthiness are used by purchasers of medicine.
• Uncertainty about medicine quality is not eliminated but managed.

## 1 Introduction

### 1.1 Uncertainty in medicine quality

*James, a 22-year-old high-school graduate, lives in in a village in southern Ghana, some 12km from the nearest clinic. He is complaining about the local drugstore, which is rarely open because the owner is busy working on his farm. The drugstore is poorly stocked and some medicines have expired. The alternative, when the situation is urgent, is to buy from itinerant peddlers who sell medicines at the weekly market and door-to-door. However, James distrusts the peddlers: he doesn't know where their medicines come from and, despite their ‘sweet words,’ he suspects they are not very knowledgeable. James always checks the expiry date, although he has heard that unscrupulous dealers repackage expired medicines or amend the date. He also checks the manufacturer's details, comparing brand numbers on the external and internal packaging. For unpackaged pills, James examines the markings on the medicine itself (‘paracetamol should have PC’), and rubs the pill between his fingers, explaining, ‘If it crumbles, you know it's old.’ He is suspicious of very cheap medicines and tries to avoid those purporting to come from India or China. Ultimately, though, James' options are limited. Unless he can find the bus fare to town, he has do make do with what is locally available.* [Author's field-notes, 17/5/2016]

James’ concerns are well-founded given the global proliferation of poor-quality medicines that have either been deliberately falsified, manufactured in substandard conditions with insufficient quality control, and/or have deteriorated post-manufacture (Hamilton et al., 2016; Tremblay, 2013; WHO, 2017a). The prevalence of poor-quality medicines is notoriously difficult to ascertain, and estimates are often disputed (Almuzaini et al., 2013). A recent comprehensive review by the World Health Organisation reported that over 10 percent of medicines in low and middle-income countries (LMICs) contain too little active ingredient, with antibiotics and anti-malarials particularly vulnerable (WHO, 2017a, 2017b).

Ghana has relatively well-functioning regulatory systems compared with other countries in the region. However, as interviewees from the Ghana Food and Drugs Authority (GFDA) told us, they remain under-resourced and lack the capacity to control effectively the supply of poor-quality medicines, especially beyond major urban centres. For example, when we interviewed them they had eight staff to do all the inspections in Central Region which has a population of over 2.2 million (Ghana Statistical Service 2013). Recent studies have reported that 20–40 percent of anti-malarials and antibiotics sold in official outlets in Ghana fail quality standards (<= 80% of active ingredient: Kaur et al., 2016; Fadeyi et al., 2015; Tivura et al., 2016). Globally, poor-quality anti-malarial and antibiotic medicines are thought to be responsible for more than 100,000 under-five deaths annually, concentrated in Sub-Saharan Africa (Renschler et al., 2015; WHO, 2017b), in addition to risks of accelerated antimicrobial resistance that arise through persistent under-dosing (Dondorp et al., 2009; Newton et al., 2016; Brock et al., 2018; Sharma et al., 2017).

The WHO and other agencies have responded with efforts to strengthen national regulatory capacity and reporting (Hamilton et al., 2016; WHO, 2017a), while advising customers to source medicines only from ‘trusted and licensed outlets’ and to scrutinise medicines carefully before purchase ( Box 1). However, as James' experience illustrates, sourcing from ‘trusted and licensed outlets’ is not always an option, while the advice to discuss ‘adverse reactions’ with a ‘pharmacist, doctor or other healthcare professional’ requires a level of access that is unavailable for most Ghanaians. Moreover, as acknowledged in the official advice, it is extraordinarily difficult to distinguish by eye a good-quality medicine from a poor-quality one even for a trained pharmacist, let alone a ‘lay’ purchaser.

Box 1 **WHO guidance on identifying a substandard or falsified medical product**

Some falsified medical products are almost visually identical to the genuine product and very difficult to detect. However, many can be identified by:

• examining the packaging for condition, spelling mistakes or grammatical errors;
• checking the manufacture and expiry dates and ensuring any details on the outer packaging match the dates shown on the inner packaging;
• ensuring the medicine looks correct, is not discoloured, degraded or has an unusual smell;
• discussing with your pharmacist, doctor or other healthcare professional as soon as possible if you suspect the product is not working properly or you have suffered an adverse reaction; and
• reporting suspicious medical products to your National Medicines Regulatory Authority.

Alt-text: Box 1

In this context, we ask how consumers in Ghana manage uncertainty in medicine quality, and how they come to trust *enough* to purchase and ingest a medicine. We focus on private-sector retail outlets (formal and informal), where the majority of people in Ghana (and other LMICs) source essential medicines, where regulation is usually patchiest, and where the ‘trust problems’ are greatest, since access to medicines is unmediated by a clinician. We begin by outlining some key features of private-sector pharmaceutical supply in Ghana and of medicine transactions in general, situating our work within a small but growing body of research on trust in healthcare in high-risk, low-regulation environments beyond the Global North.

Source: http://www.who.int/mediacentre/factsheets/fs275/en/Accessed on 05/06/2019.

### 1.2 Private sector medicine outlets and supply chains in Ghana

In Ghana (as in many other LMICs), private sector retail outlets constitute the primary source of essential medicines for most of the population. Although Ghana's National Health Insurance Scheme (NHIS) was intended to facilitate access to primary healthcare and essential medicines, in 2016, about 60 percent of the population were uninsured and thus obliged to pay out-of-pocket for essential medicines sourced predominantly from the private sector (Alhassan et al., 2016; Vandam, 2016). Medicine shortages and stock-outs at government health facilities mean that even those covered by NHIS have to resort to private-sector outlets for essential medicines, where availability tends to be better (Mackintosh and Mujinja, 2010; Palafox et al., 2014; Nicholson, 2013; Seidman and Atun, 2017). Moreover, especially in rural areas, the distance and cost of travelling to the nearest government-sector health facility can be prohibitive, and medicines may well be out of stock there.

Ghana has a huge variety of ‘private-sector outlets’, operating with varying degrees of regulation, legality and formality. These range from large, up-market, licensed pharmacies in city centres, to smaller over-the-counter (OTC) medicine stores (commonly known as ‘drug stores’ or ‘chemical shops’), to grocery stores selling basic medicines and itinerant peddlers like those in James' village (Hampshire et al., 2011, 2015). The informal sector in Ghana is smaller than in neighbouring francophone countries (Baxerres and Le Hesran, 2011); nonetheless it is estimated that unregulated drugs comprise 10–20 percent of all medicines in circulation (Seiter and Gyansa-Lutterodt, 2009). As Baxerres (2014) notes, medicines sold in informal outlets are not necessarily of poor quality, but they fall outside the scrutiny of Food and Drugs Authorities.

About 30 percent of Ghana's medicine supply is manufactured locally, with the remainder imported mostly from India and China (Akomea et al., 2014). Ghana's importation and distribution channels have been characterised as chaotic, with weak distribution networks and fragmented competition (McCabe et al., 2009). Multiple actors operate at different levels – importers, distributors, wholesalers, retailers, sales reps, and many others – with significant vertical consolidation (as the same company manufactures, wholesales and retails, for example) and horizontal transfer. ‘Leakage’ of medicines from public to private sectors has been observed while other medicines move between formal and informal sectors as they criss-cross geographical and regulatory boundaries (Dizon-Ross et al., 2017). These characteristics make pharmaceutical supply chains vulnerable to penetration of poor-quality medicines and to lapses in appropriate standards of storage and transportation (Tremblay, 2013; Patouillard et al., 2010).

### 1.3 Buying medicines: an asymmetrical economic transaction

A medicinal transaction occurs when a buyer and seller exchange money for medicine. In general, the buyer assumes that the product will do what it says it will do on the packet, and that the seller will act with integrity, dispensing a product appropriate to the condition. However, uncertainty about medicine quality creates three important conditions.

First, sellers usually know more about medicine quality than buyers do. The difficulties of distinguishing an effective medicine from an ineffective one means that (unlike some other commodities), neither buyer not seller can necessarily be certain about quality (Mackintosh and Mujinja, 2010; Mackintosh et al., 2018). However, retailers usually have more information on the source of a medicine than a buyer (albeit not necessarily of the full supply chain). Even retailers without formal qualifications typically have more knowledge and experience than the public, enabling them to make more informed decisions.

Second, the consequences of getting it wrong are typically greater for buyers, who risk their health (or that of a loved one), than for sellers. The latter may risk regulatory penalties and/or reputational damage for selling poor-quality medicines, but in practice this very rarely happens in Ghana, owing to poor detection/reporting systems and an unwillingness to report suspect products/people (discussed below). This asymmetry of risk places a much larger burden on the buyer to make the right decision than on the seller.

Third, in Ghana, pharmaceutical medicines are typically bought when someone is actually unwell (unlike herbal medicines, which tend to be used prophylactically; see Hampshire et al., 2013). Being unwell (or seeing a loved one unwell) can convey a sense of urgency to procure treatment that puts the customer in a position of structural disadvantage vis-à-vis the seller, perhaps increasing her susceptibility to the seller's interests (for example, shifting a more expensive or profitable product).

Private-sector medicine transactions in situations of relatively low regulatory capacity and enforcement thus occur within a context of uncertainty, informational asymmetry and buyer vulnerability. While most medicine retailers may be competent and motivated by pro-social desires to help, this cannot always be assumed; moreover, retailers may also not have enough information to be certain about a medicine's quality. Uncertainties, informational asymmetries and vulnerabilities can also pervade transactions higher up supply chains, as retailers make decisions about procurement, with less information on a medicine's quality and provenance than their suppliers (Chaudhuri et al., 2010). We ask how, in a practical sense, buyers of pharmaceutical medicines in Ghana manage this uncertainty.

### 1.4 Trust in healthcare

Kenneth Arrow (1963) and Richard Titmuss (1970) noted the uncertainties and asymmetries often inherent in healthcare. Trust becomes most salient where uncertainties are greatest. As Giddens (1990: 33) stated, ‘There would be no need to trust anyone whose activities were continually visible and whose thought processes were transparent, or to trust any system whose workings were wholly known and understood.’ It is generally accepted in the sociological literature that trust is granted to those we find trustworthy, i.e. those who demonstrate competence and integrity (see Cook and Gerbasi, 2009, for a review). This formulation has underpinned the majority of research on trust in healthcare, focused mostly on doctor-patient relationships in ‘Western’ settings (e.g. 2008; Calnan and Rowe, 2008; Mechanic and Meyer, 2000; Meyer and Ward, 2013), and/or surveys of generalised trust in (North American and European) healthcare institutions (see reviews by Gilson, 2003; Cook Karen and Stepanikova, 2009; and critiques by Brhlikova et al., 2011; Ozawa and Sripad, 2013). In this work, trust and trustworthiness are understood in the context of robust regulatory institutions and meaningful choice.

In recent years, research has increasingly included resource-poor contexts, where inadequate service provision and financial barriers may severely constrain choice (e.g. Birungi, 1998; Gilson et al., 2005; Russell, 2005; Ozawa and Walker, 2011; Tibandebage and Mackintosh, 2005; Hampshire et al., 2017; Ackatia-Armah et al., 2016). This work has highlighted the importance of social relationships and mutual understanding between healthcare providers and those seeking care (Gilson, 2003; Tibandebage and Mackintosh, 2005:1385; Brhlikova et al., 2011; Ecks and Basu, 2014). In settings where the risks and uncertainties may be extraordinarily high (Rodrigues, 2016), some researchers have questioned the premise that trustworthiness underpins trust. They have highlighted the importance of *living with* rather than seeking to eliminate uncertainty (Samimian and Rabinow, 2015; Widger, 2017; Zin, 2016), and the need sometimes to “trust the untrustworthy” (Haas, 2016; Tibandebage and Mackintosh, 2005).

Following these approaches and referring to a transaction between individuals, we define the act of trusting as the *expectation* that purchasing or taking a medicine will do more good than harm, even if the benefit is marginal. Below, we explore the cognitive and affective bases underpinning the formation of trust in this type of interaction and the *limits* of these processes in managing ‘deep’ uncertainty over medicine quality.

## 2 Methods

This paper draws on nine months' fieldwork (May 2016–February 2017) conducted in Ghana by the authors and research assistants. Fieldwork was conducted principally in Ghana's Central Region: Cape Coast, the Regional capital, and several smaller settlements, with additional fieldwork in Accra. We present data from 200 semi-structured interviews with retailers and customers, accompanied with ethnographic observations, at a range of private-sector medicine outlets; a further 20 ‘non-transaction interviews’ plus 20 key-informant interviews with regulators in Cape Coast and Accra.

Medicine outlets were selected purposively, to reflect the observed variation in type, urban/rural location, and proximity to other (competing) outlets. The sample comprised: pharmacies (N = 35), licensed over-the-counter (OTC) medicine shops (N = 80), general stores stocking medicines (N = 27) and marketplaces (N = 58). Many outlets sold both pharmaceutical and herbal medicines, but this paper focuses on pharmaceuticals only.

In each outlet, the researcher began by requesting the retailer's permission to be in the store (or stand by the market stall) and explained that we were interested in the medicines customers were buying and why they were choosing these particular products. Researchers made detailed observational notes on the store (general conditions, set-up, range of medicines sold, etc.) and interviewed the retailer(s) about the business (who owned the store, how long it had been operating, stocking decisions, etc.). Every interaction with customers was observed and detailed notes taken on the conversation and what (if any) medicine was sold.

Immediately after any transaction, we requested an interview with both customer and retailer about the purchase. These ‘post-transaction interviews’ were open-ended and conversational. Customers were prompted to explain why they had chosen to buy *that particular medicine* from *that particular outlet*, and what kinds of considerations influenced their purchasing decision. Retailers were asked about why they had sold that particular medicine, their discussion with the customer prior to the purchase, and their views on medicine quality. Interviewers were careful not to ask about trust specifically but to probe whenever the interviewee brought up issues of trust or uncertainty. On average, interviews lasted 30–45 min, depending on interviewees' willingness and availability.

The combination of interview and observational data enabled us to cross-check and verify interviewees' accounts and practices. In order to mitigate possible observer effects (for example, a retailer being more attentive or following dispensing protocols more strictly than usual), researchers spent two or 3 day at each outlet, remaining as unobtrusive as possible. We observed a great many ‘unconventional’ dispensing practices that lead us to believe that our presence did not affect behaviour very much but, of course, this remains a possible limitation of the study.

Additional ‘non-transaction interviews’ (N = 20) were conducted in smaller rural settlements with no functioning medicine outlet. Using purposive sampling, these interviews enabled us to capture the experiences of people whose access to medicines was particularly limited and who may not have purchased pharmaceutical medicines recently. In practice, however, it was rare to encounter someone who did not purchase or consume pharmaceutical medicines altogether: decisions were mostly about when, what and where, not *whether*.

Data analysis followed the principles of Grounded Theory. Theoretical insight emerges from the data through an iterative process of close-reading, coding and testing of nascent hypotheses through subsequent fieldwork (Corbin and Strauss, 2015). In the first cycle, data were coded descriptively according to type of medicines bought/sold, outlet type, and considerations influencing purchasing/retail decisions. In the second cycle, the codes were then condensed and integrated into broader and more coherent categories and themes that led to identification of the mechanisms discussed in this paper. The quotations throughout this paper are illustrative of the wider sample's responses.

Ethical approval was granted by Oxford University's Social Sciences & Humanities Inter-Divisional Research Ethics Committee (UK); Durham University's Anthropology Ethics Committee (UK); and the University of Cape Coast Institutional Review Board (Ghana). All names have been changed and some other details modified to protect anonymity. Informed consent was sought verbally from each participant before commencing data collection (observations and interviews). We were mindful that some customers might be anxious to leave quickly after purchasing a medicine; consequently, some interviews were short and/or curtailed, but we always put the needs/wishes of the interviewee first. Most people, however, were eager to share their experiences, generating a rich dataset.

## 3 Results

### 3.1 Perceiving and experiencing uncertainty in medicine quality

Luhmann (2017) has argued that trust-based decision-making occurs only if there is an awareness of risk or uncertainty of outcome. Our interview data indicated very clearly that research participants, in both rural and urban areas, perceived a high degree of uncertainty about the quality and efficacy of the medicines available in private-sector outlets.

First, research participants talked about **‘fake’ medicines**, deliberately manufactured with little/no active ingredient for financial gain – a perception fuelled by reports on television, radio and local public address systems. ‘Fake medicines’ were believed to be ineffective, with the potential to cause significant harm:

‘*Once I bought a medicine and, when I poured it, it had such a bad smell. When I took it, my body reacted [badly] to it – it was a really bad experience. I checked again and it had not expired. I believe it was a fake medicine. […] I was so terrified that I could have died*.’ [48y man, rural]

Expired or otherwise **deteriorated medicines** were a second source of anxiety, especially in rural areas where medicines are said to ‘hang around’ on shop shelves and/or be stored incorrectly. Many research participants worried about taking ‘spoiled medicines’ which were widely seen to be ‘*poisonous and harmful*’, [25y man, rural].

Finally, some consumers were worried about getting the **wrong medicine** (irrespective of inherent quality). In resource-constrained contexts like Ghana, where diagnostic equipment is scarce, treatment is typically symptom-based and diagnosis by treatment (‘trying out’: Whyte, 2005:249) remains the norm. (Even Rapid Diagnostic Tests for malaria were rarely used by our research participants.) The risk of misdiagnosis and mis-prescription is therefore high, particularly for aetiologically-distinct diseases with similar symptoms (e.g. malaria, typhoid and urinary-tract infections). Some interviewees also spoke about unscrupulous vendors who would sell unnecessary or inappropriate medicines, just to ‘make business.’

A perception of uncertainty about medicine quality extended to some retailers we interviewed, although they rarely shared their doubts with customers. For example, Joseph, an Accra-based pharmacist, said he struggled to differentiate between ‘genuine’ and ‘fake’ medicines sold by local distributors (an issue we return to later). Others worried that medicines might deteriorate before reaching their shops. For example, Hawa, another Accra-based pharmacist, told us that, ‘*Even when medicine [like insulin] comes packaged in ice, maybe it hasn't always been stored in ice – maybe they just add the ice for show when they are delivering it.*’

In summary, both end-user customers and retailers perceived risks and uncertainties around medicine quality and, for many, this caused significant anxiety and concern. We now turn to the question of how this perceived uncertainty is managed.

### 3.2 Mitigating uncertainty: seeking observable signs of quality

Consumers face a dilemma: they need (or believe they need) medicine to get better, but they cannot observe directly a medicine's contents or quality. Our data show that buyers make multiple assessments of the appearance of both the medicine and the retail outlet, in a manner reminiscent of the WHO advice. We identified seeking observable signs of quality as a mechanism underpinning the assessment trustworthiness.

#### 3.2.1 Checking the medicine

Buyers sought reassurance by inspecting the medicine. Younger, better-educated ones like James said that they checked medicine expiry dates before purchasing any medicine. Some showed us how they scrutinise the packaging and information leaflet for indicators of official certification, checking for inconsistencies indicating possible fraud. Customers with lower levels of literacy scrutinised the colours and images on packaging: *‘If I am given something with a different shape, package or colour, I will suspect it is fake.*’ [34y woman, urban].

For unpackaged medicines, widely sold in smaller and informal outlets, the appearance, feel and taste of the medicine become important. Poor-quality pills were generally thought to have a faded appearance and/or a crumbly texture:

‘*For medicines that I have been using for a very long time, like paracetamol, I press to see how hard or soft it is. If the medicine is very hard, that means it has not expired but if it is soft that means it has kept long at the shop – not necessarily that it has expired but I need to be careful with it*. […] *Life is the most precious gift so I don't joke with it for me or my family*’ [22y woman, rural]

Interviewees sought to reduce uncertainty and maximise ‘potency’ by preferring to buy medicines apparently originating from Europe or North America rather than ‘local’ ones (Mackintosh and Mujinja, 2010; Mujinja et al., 2014). Interviewees tended to be particularly suspicious of certain Asian-manufactured generics:

‘*I buy medicines from any country except China. China is well noted for fake things so the likelihood of them producing fake medicines is also high; I don't trust Chinese products*' [27y woman, peri-urban].

Buyers generally believed that price was a good proxy for quality: ‘*Usually, the good medicines are expensive – the better the medicine, the higher the price*’ [48y woman, peri-urban]. This interviewee told us that she avoided the cheapest products where possible but she, like many, could not afford an expensive branded medicine and so was buying a mid-range anti-malarial when we met her.

Similar strategies were used by retailers attempting to manage uncertainty in medicines they were purchasing, and concomitant risks (to their businesses and/or their customers' wellbeing). Several described in detail the checks that informed their purchasing decisions; just like their customers, these included scrutinising external and internal packaging, opening packets and assessing the colour and texture of medicines, etc. Also, like their customers, they were wary of very cheap medicines; as one OTC shop owner put it, ‘*When I know the price of a medicine and then someone comes with one that is extremely cheap, I become suspicious*.’

#### 3.2.2 Scrutinising the outlet

Again in line with WHO advice, many interviewees tried to reduce uncertainty by sourcing their medicines from ‘trusted and licensed outlets’, preferring licensed pharmacies because: “*Their medicines are more likely to be purchased from authorised manufacturers so safety is assured … I believe they will have done their checks well.*’ [26y woman, urban]. OTC medicine shops and general stores were often thought to stock poorer quality medicines: ‘*When I see those chemical [OTC] shops, my feeling is that they could be engaging in some fraudulent stuff and their medicines may be fake*,’ said one man (31y, urban).

Customers generally favoured well-established, popular outlets, reasoning that such establishments would not wish to risk reputational damage. Size and location also mattered: interviewees generally believed that large, centrally located establishments would find it more difficult to get away with selling dubious medicines than smaller, more ‘hidden’ ones:

‘*Bigger shops, the probability they will sell fake medicines is very minimal compared to ones in the village. They have invested a lot – it is a sign they have in mind to do good business.*’ [22y woman, rural]

Some also assumed that large shops have a more rapid turnover of stock, thereby reducing the risk of medicines deteriorating. For similar reasons, prospective customers scrutinised the general conditions and medicine storage facilities in a shop: ‘*It is a big pharmacy and their medicines are stored in a very good condition, at the right temperature so that the potency of medicines is not compromised*’ [24y woman, rural]. Buyers also felt more confident in the abilities of smartly dressed, professional-looking staff who spoke knowledgeably in a pre-sales discussion:

‘*You can talk to them to see that the person has been trained. If I talk to you and see that you have not been trained, I will not buy from you because it can be dangerous. I may have headache and you give me medicine for stomach-ache. You can tell by the detail they go into when they talk whether they have been trained*’ [20y man, rural].

### 3.3 Addressing informational asymmetry

Positioned at the end of the supply chain, buyers have less information about the quality and provenance of the medicine than those operating ‘higher up’. Addressing this informational asymmetry, through garnering additional information from other actors, was a second mechanism underpinning many buyers' assessments of trustworthiness.

#### 3.3.1 Reputation and popularity

Faced with a lack of reliable information about quality, many people took into account a medicine's reputation and popularity. As well as drawing on experiences of those within their personal social networks, many interviewees paid attention to media coverage, especially popular radio call-in shows:

‘*Most medicines that come to the market have advertisements, and you can listen to the advert. If they are advertising a lot and people are calling in – at least three people, say – to testify that it has worked for them, you know the medicine is very good*.’ [20y man, rural]

Length of time on the market was also considered a proxy for quality, although this interviewee added the caveat: ‘*There are times when a medicine becomes so popular and there is a higher demand for it, some fraudulent people capitalise on it to produce some fake ones in the market*.’ [28y man, rural]. This reasoning also extends to buyers repeatedly purchasing the same brand of medicines and rejecting alternative brands or even the same brand packaged differently. Some came to the shops with empty packets, bottles and blister packs asking for exactly the same thing (see also Ecks and Basu, 2014). The habitual practice of taking the same medicine reduced feelings of uncertainty based on the reasoning that if it worked before, then it will probably work again. This “embodied experience” (Rodrigues, 2016) provided interviewees with crucial information about the ‘compatibility’ between the medicine and the person (Whyte et al., 2002).

Supplier reputation is also important to retailers, especially when sourcing from local manufacturers. One OTC retailer explained his choice:

‘*Because they are nationwide acclaimed pharmaceutical companies. […] All their drugs are FDA certified, so they are of high quality. There has never been a case where they were involved in any fraudulent activity like selling fake drugs*.’

#### 3.3.2 Seeking information from staff

During our ethnographic work, we observed buyers seeking to bridge the information gap by asking the sellers to recommend medicines and for advice on storage and dosage. These conversations happened more often in pharmacies but not exclusively so. Interviewees spoke passionately about their desire for information and their frustration at being served by those with little experience or knowledge of the medicines they were selling. As one male customer put it, ‘*A small boy cannot talk and explain things to me*!’ Another said of his village OTC shop: *‘He leaves the shop for the wife to take control. The wife does not know much about the drugs. At times if you mention the drug, she will ask you to come in and show her*’ [40y, man, rural].

### 3.4 Buyer vulnerability and the limits of cognitive-based assessment

Despite assiduous attempts on the part of many buyers to mitigate uncertainty, many recognised the inadequacy of using observable proxies to determine medicine quality – and their ability to act on this information. Many of our interviewees expressed deep concerns about their vulnerability: “*At times I fear for our lives”* said one man.

For example, an expiry date can only reveal that, according to the label, the medicine is in-date. It gives no information about ingredients, production quality, storage conditions, or indications for particular symptoms. Expiry dates, like signs of official accreditation, can be falsified:

‘*These days in Ghana, anything is possible. You just have to pay something to corrupt officials and they'll do whatever you want. […] A person can just take a certificate from someone else and copy it – nobody would know*.’ [27y man, rural]

For many, the opportunities to act on assessments of (un)trustworthiness are also limited. For example, James is unemployed: with minimal disposable income, finding the bus fare to travel to town is difficult. Even if he does not really trust them, he has to make do with the medicines available locally. As the Assemblyman of James’ village noted:

‘*People use the peddlers even though their drugs are more likely to be expired than the drug store because they are cheaper and accessible – they come to people's homes. If it's not for the peddlers and the drug store is shut, they have to go to town, which is expensive*.’

Even the “embodied experience” (Rodrigues, 2016) of taking medicine could not fully close the information gap. The relationship between taking a medicine and getting better is not straightforward. Widespread polypharmacy, combined with placebo effects and the natural progression of an illness make it difficult to ascribe therapeutic failure to medicine quality. There is also a widely-held belief in Ghana that medicines are ‘personal’ – particular medicines work for particular people and not for others (Whyte et al., 2002), further stymieing inferences about a medicine's trustworthiness, even after the event.

### 3.5 Blind trust?

In contrast to the assiduous checking and information seeking observed in many cases, other participants bought medicines very quickly, completing the transaction in seconds. These buyers did not scrutinise medicines or ask searching questions; nor did they appear to be any less vulnerable than those who did.

Other researchers have grappled with the puzzle of why individuals might appear to trust ‘blindly’, even when they may have good reason to doubt. Based on work in post-socialist Slovakia, Torsello (2008) argued that if conditions are sufficiently bad, it can make sense to trust people *known (or suspected) to be untrustworthy*, because the consequences of *not trusting* can be worse than trusting and being betrayed. Haas (2016) identified a different reason for ‘trusting the untrustworthy’ in Mongolia, where the act of trusting is a moral one, bestowing a moral imperative on the recipient to act in a trustworthy manner.

When someone is acutely sick, there are strong practical, economic and emotional pressures to act. With limited options, buyers may have to accept a lower threshold of trustworthiness than might otherwise be the case. As Gambetta (1988:223) put it, ‘We may have to trust *blindly*, not because we do not or do not want to know how untrustworthy others are, but simply because the alternatives are worse’. This is not *quite ‘*dependency (Meyer and Ward, 2013) or ‘reliance’ (Hart, 1988): there is always an option to forego the pharmaceutical sector altogether and turn to herbal preparations, faith healing or do nothing at all and hope for the best. But, where pharmaceutical transactions do occur, careful scrutiny and probing questions may be pointless if the only option is to buy whatever is stocked in the local store.

### 3.6 Vulnerability and socially embedded trust

Over half of our interviewees were making a *repeat* visit to the medicine outlet, often frequenting the same store over many years. Their purchases had become *socially embedded*: inter-personal exchanges drawing on both economic and social ties, reinforced over time. In these cases, buyers were attempting to alleviate their vulnerability by invoking an *affective* basis for trust, entailing ‘emotional bonds and obligations generated through repeated interaction, empathy and identification with the other's desires or intentions' (Gilson, 2003:1456). For example, Efua, an elderly woman based in Cape Coast, trusts a particular dispenser who has worked at the OTC shop for a long time and whom she assumes will have acquired considerable knowledge. More important, though, is the *personal* relationship they have developed: if she happens to be unavailable, Efua prefers to wait or return another time rather than see someone else.

In other cases, social connections between buyer and seller *precede* the economic relationship and emanate elsewhere: through kinship, enduring friendship or connections from church, school or another third party. Once the relationship is established, and repeated transactions have occurred, the impetus to address uncertainty and informational asymmetry diminishes:

‘*I don't check anything on the medicine before buying it from this shop. I just come to buy. She [seller] is good, honest so I know she will not sell bad drugs to me.*’ [38y man, urban]

Where trust is socially embedded, buyers are inclined to give the retailer the benefit of the doubt, even when presented with possible evidence to the contrary: *‘If it is like this place where I have been buying all the time and never had any problem, I will not be disappointed [if a medicine turned out to be ineffective] because it may be a mistake.*’ [35y woman, urban]. Trusting, even when there is reason to doubt, reinforces the relationship and intensifies the moral obligation to be trustworthy (Haas, 2016). Having established a relationship, it only makes sense to quit if the actor believes strongly that they could do better elsewhere, especially given the investment of time and effort needed to build a new relationship. Similar considerations underpin the purchasing decisions of some retailers. Constrained by resource limitations and aware that they cannot eliminate uncertainty and informational asymmetry, it makes sense to stick to a single supplier with whom they have established a relationship.

Where trust is socially embedded, and/or where it emanates from feelings of desperation, it may not just be *unnecessary* (or pointless) to scrutinise and ask questions; it may be *counterproductive*. Where the buyer has no option than to buy what is available, it may be psychologically easier *not* to look too carefully (Gambetta, 1988: 224). Moreover, where purchases are embedded in strong economic and social ties, suspicious behaviour (excessive checking or asking too many probing questions) may undermine moral and social relations and damage a long-term relationship with future negative consequences:

“*You see the truth is that the man is good but at times he drinks, so when he drinks his mood swings … But I am not just his customer, we also attend the same church. If I don't buy from him, he will tell our pastor. Moreover as a fellow church member, I have to buy from him so that his business will boom. I don't have any option than to buy from him.*” [40 yr, man, rural].

## 4 Conclusion

In this paper, we show how Ghanaians in our sample attempt to assess the trustworthiness of medicine at the point of transaction in a context of perceived high uncertainty and relatively low regulation. In the opening vignette, James tries to mitigate uncertainty by scrutinising the medicine for observable signs of quality – a widespread practice among our sample. Others sought to close the information gap by seeking as much information as possible about medicines before purchasing.

However, these cognitive-based calculations of trustworthiness are of limited utility, especially in situations of extreme poverty, constraint or desperation, where the choice may be to take whatever is available or do nothing at all. To deal with this, some interviewees embedded their transactions within enduring social and economic relationships. In these cases, trust has a relational and affective basis, which risks being undermined by the cognitive-based approaches of scrutiny and information seeking. In Ghana, such relationships form the basis of many commercial transactions (see, for example, the work of Lyon, 2000, Lyon, 2006 on tomato farmers). Engaging in repeat transactions over long periods gives each party the opportunity to learn about the other, and the expectation of future interactions produces a strong incentive to cooperate.

Our analysis concurs with the work of others who highlight the importance of affective bases for forming trust when cognitive mechanisms are inadequate (Gilson, 2003; Tibandebage and Mackintosh, 2005: 1385; Brhlikova et al., 2011; Ecks and Basu, 2014). Of course, the cognitive/affective distinction is not a simple and stable binary; nor does purchasers’ behaviour fall unambiguously into one category or another. In reality, many people base their assessments of trustworthiness on a fragile balance of the two approaches that may shift over time as circumstances change. Nonetheless, these distinctions have heuristic value, helping us to understand how people in Ghana manage the unenviable situation of transacting and consuming medicines when uncertainties and stakes are very high.

However, even the affective relationships cannot *eliminate* uncertainty: they can only make it more *manageable* (Samimian and Rabinow, 2015; Widger, 2017; Zin, 2016). Without expensive equipment and expert knowledge, no one can accurately distinguish a high-quality medicine from a poor-quality one. Unlike the tomato farmers described by Lyon, 2000, Lyon, 2006, who know with certainty about the quality of their own products, medicine sellers – from itinerant peddlers to high-end licensed pharmacists – cannot be certain. No matter how strong and embedded the bond of trust, the retailer-customer (or wholesaler-retailer) relationship is just one link in complex, interwoven global supply chains, where no one has full information (Tremblay, 2013; Mackintosh et al., 2018, 2018). Just one ‘weak link’ can result in a poor-quality medicine as Joseph, the pharmacist, explained:

‘The issue of sourcing medicines here is very complex because there are not specific supply chains. There are so many wholesalers. […] The combination of a complex supply chain and lack of regulation makes it more or less impossible to be sure about medicine quality.’

We should not forget why it is that medicine sellers and buyers in Ghana (and many other LMICs) have to make do with such imperfect strategies. Elizabeth Cooper (2015) reminds us that uncertainty is primarily not a ‘cultural’ issue but a political and economic one, rooted in inequitable control over resources, including knowledge. In Ghana, the uneven distribution of knowledge and resources renders some people more vulnerable than others to potential harms resulting from poor quality medicines. At a global level, deep geopolitical inequities that, for decades, have been mapped onto access to essential medicines (Greene, 2011) re-emerge in the form of new risks and uncertainties around medicine quality. While our focus has been the pragmatic management of uncertainty, this must not detract from the need to recognise and address the underlying power inequities that perpetuate this situation.

Practically, there have been many attempts by national and international agencies to stem the global circulation of poor-quality medicines, addressing both the demand side (through tighter regulation, better reporting mechanisms and more effective enforcement) and the supply side (through public and clinician education) (Hamilton et al., 2016; WHO, 2017a). However, the problem persists. We suggest that policy-makers and regulators have paid insufficient attention to *what actually happens* when medicines are transacted and *how people come to make the decisions they do* – often in less-than-ideal circumstances. There are clearly no quick fixes: the large sums of money at stake, combined with under-resourced regulatory systems and high unmet demand for essential medicines, make the problem of poor-quality medicines a particularly intractable one. However, a clear understanding of what actors are *actually* doing and thinking, rather than relying on untested assumptions, is a good starting point. This will require careful ethnographic research, not just at the end-points of supply chains but all the way up as actors navigate uncertainty with different motivations, constraints and exigencies.
